# TPP-Thiazole Derivatives Ameliorate Psoriasiform Inflammation by Glycolysis Inhibition

**DOI:** 10.3390/molecules31060982

**Published:** 2026-03-15

**Authors:** Xinwei Meng, Ci-An Cheng, Zhirui Zhang, Siying Qu, Anqi Zhang, Yang Zhang, Jinxin Gu, Hanwen Zhang, Keyue Ding, Lei Fu, Mengchen Lu, Daiyun Huang, Yixue Qiao

**Affiliations:** 1 Wisdom Lake Academy of Pharmacy, Xi’an Jiao Tong Liverpool University, Suzhou 215123, China; 15161882921@163.com (X.M.); cian.cheng23@student.xjtlu.edu.cn (C.-A.C.); zhirui.zhang24@student.xjtlu.edu.cn (Z.Z.); siying.qu22@student.xjtlu.edu.cn (S.Q.); anqi.zhang22@student.xjtlu.edu.cn (A.Z.); jinxin.gu02@xjtlu.edu.cn (J.G.); h73135z342136@126.com (H.Z.); keyue.ding22@student.xjtlu.edu.cn (K.D.); lei.fu@xjtlu.edu.cn (L.F.); 2 School of Pharmaceutical Sciences, Shanghai Jiao Tong University, Shanghai 200240, China; yang.zhang6@sjtu.edu.cn; 3 Department of Medicinal Chemistry, College of Pharmaceutical Sciences, Soochow University Medical College, Suzhou 215123, China

**Keywords:** TPP-thiazole derivatives, psoriasiform inflammation, mitochondria, glycolysis inhibition, immune-metabolic regulation

## Abstract

Psoriasis, a chronic inflammatory skin disease, is driven by immune dysregulation and keratinocyte hyperproliferation, with current biologics facing limitations. Emerging evidence points to mitochondrial dysfunction and a pathological shift to aerobic glycolysis as core disease drivers. Here, we report that MitoFu-O, a novel mitochondria-targeting TPP-thiazole derivative, effectively ameliorates psoriasiform inflammation in imiquimod-induced mice and cytokine-stimulated keratinocytes. Mechanistically, MitoFu-O acts by inhibiting pathological glycolysis, downregulating key glycolytic enzymes (HK1, GAPDH, LDHA), and subsequently suppressing the activation of pivotal pro-inflammatory signaling pathways (MAPK, NF-κB, and STAT3). Our findings establish targeted mitochondrial modulation as a potent therapeutic strategy, positioning MitoFu-O as a promising lead compound that acts upstream of cytokine signaling by normalizing the metabolic reprogramming fundamental to psoriatic pathogenesis.

## 1. Introduction

Psoriasis is a chronic, immune-mediated inflammatory skin disease characterized by aberrant cytokine signaling that disrupts epidermal homeostasis, leading to keratinocyte hyperproliferation and altered differentiation [1,2,3,4]. Psoriasis has a global prevalence of 2–3% and is frequently associated with systemic comorbidities, including psoriatic arthritis in approximately 30% of patients. The incurable, relapsing nature of the disease imposes a significant multidimensional burden on patients, highlighting the imperative for management strategies that integrate control of both cutaneous and systemic inflammation [5,6,7,8].

The pathogenesis of plaque psoriasis is driven by T-cell activation and the subsequent overproduction of key pro-inflammatory cytokines, including TNF-α, IL-17, and IL-23 [9,10]. This cytokine milieu directly induces the abnormal proliferation and impaired differentiation of keratinocytes, culminating in epidermal acanthosis (thickening) and the development of characteristic scaly plaques. Furthermore, disease progression is exacerbated by concurrent angiogenesis and skin barrier dysfunction [11]. Mitochondrial dysfunction is increasingly recognized as a key contributor to psoriasis pathogenesis [12].

Mitochondrial dysfunction in psoriatic keratinocytes, characterized by bioenergetic failure and excessive ROS production, propagates inflammation via pathways such as NF-κB. This self-perpetuating cycle establishes mitochondria as a key contributor to psoriasis and a viable therapeutic target [13,14,15].

Although biologics against key cytokine pathways (IL-17, IL-23, etc.) represent a therapeutic advance, they are limited by cost, unpredictable response, tachyphylaxis, and immunosuppressive sequelae [16,17]. A significant shortcoming of existing modalities is their inadequate address of comorbid metabolic disturbances and oxidative stress. This unmet need is driving the exploration of novel interventions that holistically target cellular bioenergetics and inflammatory networks, with mitochondrial function emerging as a pivotal regulatory node [18,19,20,21].

Our group has developed triphenylphosphonium (TPP)-thiazole derivatives [22,23,24], a class of mitochondria-targeted compounds engineered for organelle-specific delivery. Their structure comprises a thiazole pharmacophore connected via a linker to a TPP cation, which ensures mitochondrial accumulation driven by the membrane potential [22,23,24]. The cell membrane potential ranges from 30 to 60 mV, while the mitochondrial membrane potential is between 150 and 180 mV. Although many small-molecule drugs can readily cross the cell membrane, they are often unable to penetrate the mitochondrial bilayer membrane to exert their effects within the mitochondria. TPP^+^ increases the molecule’s positive charge, thereby enabling efficient delivery of active small molecules into the mitochondria. Our previous work established that chronic treatment with a prototype TPP-thiazole induces mild, reversible Complex IV inhibition [25]. This moderate OXPHOS restraint reduced ROS, activated mitochondrial biogenesis and mitophagy via the AMPK/PGC-1α axis, and ultimately alleviated age-related metabolic dysfunction and inflammation in mice, demonstrating its therapeutic potential without overt toxicity.

**Figure 1:**
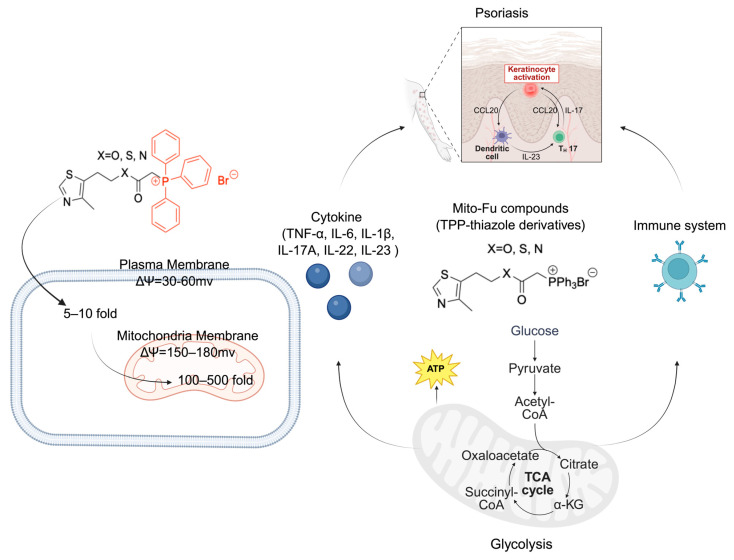
Mitochondria-targeted metabolic modulation attenuates psoriatic inflammation through keratinocyte–immune crosstalk. Psoriasis pathogenesis: keratinocyte activation triggers the release of C-C Motif Chemokine Ligand 20 (CCL20), promoting the recruitment of dendritic cells (DCs) and subsequent activation of the T helper 17 (Th17) axis through Interleukin-23 (IL-23) and Interleukin-17 (IL-17) signaling, resulting in chronic skin inflammation and epidermal hyperplasia. Mitochondrial targeting can be achieved by conjugating drug molecules with the TPP^+^ cation, which exploits the substantial electrochemical gradient created by the mitochondrial membrane potential (5–10 times that of the plasma membrane) to facilitate uptake into mitochondria. Within mitochondria, the tricarboxylic acid (TCA) cycle maintains oxidative metabolism and energy homeostasis, generating adenosine triphosphate (ATP) from glucose-derived intermediates, including acetyl coenzyme A (acetyl-CoA), alpha-ketoglutarate (α-KG), and succinyl coenzyme A (succinyl-CoA). Mitochondria-targeted modulation restores cellular redox balance, normalizes keratinocyte metabolism, and dampens pro-inflammatory cytokine production, thereby alleviating psoriatic inflammation and breaking the keratinocyte–immune feedback loop.

Given the established role of mitochondrial dysfunction and redox imbalance in driving psoriatic pathology, we hypothesized that targeted mitochondrial metabolic modulation with our TPP-thiazole derivatives would confer therapeutic benefits in psoriasis (Figure 1). The role of metabolism regulation in psoriatic inflammation, however, is poorly defined. Here, we investigate three optimized TPP-thiazole derivatives (MitoFu-O, S, N) to test the hypothesis that targeted mitochondrial modulation can alleviate psoriasis. By examining their impact on energy metabolism and inflammation in M5 cytokine (IL-22, IL-17A, oncostatin M, IL-1α, and TNF-α)-stimulated keratinocytes and in an imiquimod (IMQ)-induced mouse model, this work aims to establish a foundational link between metabolic reprogramming and inflammatory resolution, proposing a new strategic avenue for psoriasis treatment.

## 2. Results

### 2.1. MitoFu-O Alleviates Inflammatory Responses in the M5 Cytokine-Induced Psoriatic Keratinocyte Model

**Figure 2:**
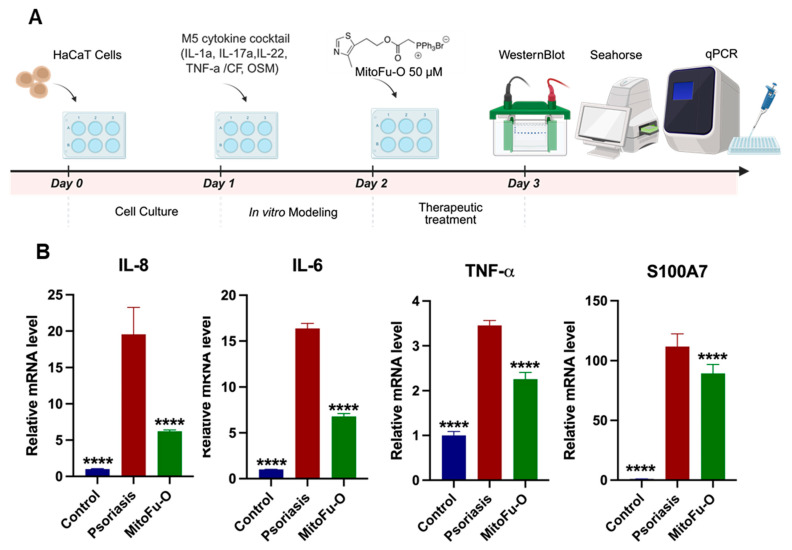
MitoFu-O alleviates inflammatory responses in the M5 cytokine-induced psoriatic keratinocyte model. (**A**) Schematic illustration of the experimental workflow for the M5 cytokine-induced psoriatic cell model and subsequent MitoFu-O treatment. (**B**) RT-qPCR analysis of inflammatory and psoriatic marker genes (IL-6, IL-8, S100A7, and TNF-α) after 24 h treatment with 50 μM MitoFu-O. Data are mean ± SEM, N = 3, **** *p* < 0.0001.

To preliminarily validate the anti-inflammatory efficacy of MitoFu-O at the cellular level, the M5 cytokine-induced HaCaT cell model was established, and the mRNA expression of key psoriatic markers was assessed by RT-qPCR (Figure 2A). Consistent with the hyperinflammatory state of psoriasis, M5 stimulation significantly upregulated mRNA levels of the potent neutrophil-chemoattractant IL-8, the pleiotropic pro-inflammatory cytokine IL-6, and the master regulator of inflammation TNF-α compared with the control group (Figure 2B). Furthermore, the expression of S100A7 (psoriasin), a damage-associated molecular pattern (DAMP) protein specifically overexpressed in psoriatic skin and crucial for its pathogenesis [26], was also markedly increased. Treatment with MitoFu-O significantly reversed this effect, leading to pronounced downregulation of mRNA expression for all these markers (Figure 2B). These data indicate that MitoFu-O effectively attenuates the transcriptional activation of critical mediators driving both inflammation and the characteristic epidermal dysfunction in psoriasis.

### 2.2. MitoFu-O, S, N Improves Psoriatic Symptoms in IMQ-Induced Mouse Model

**Figure 3:**
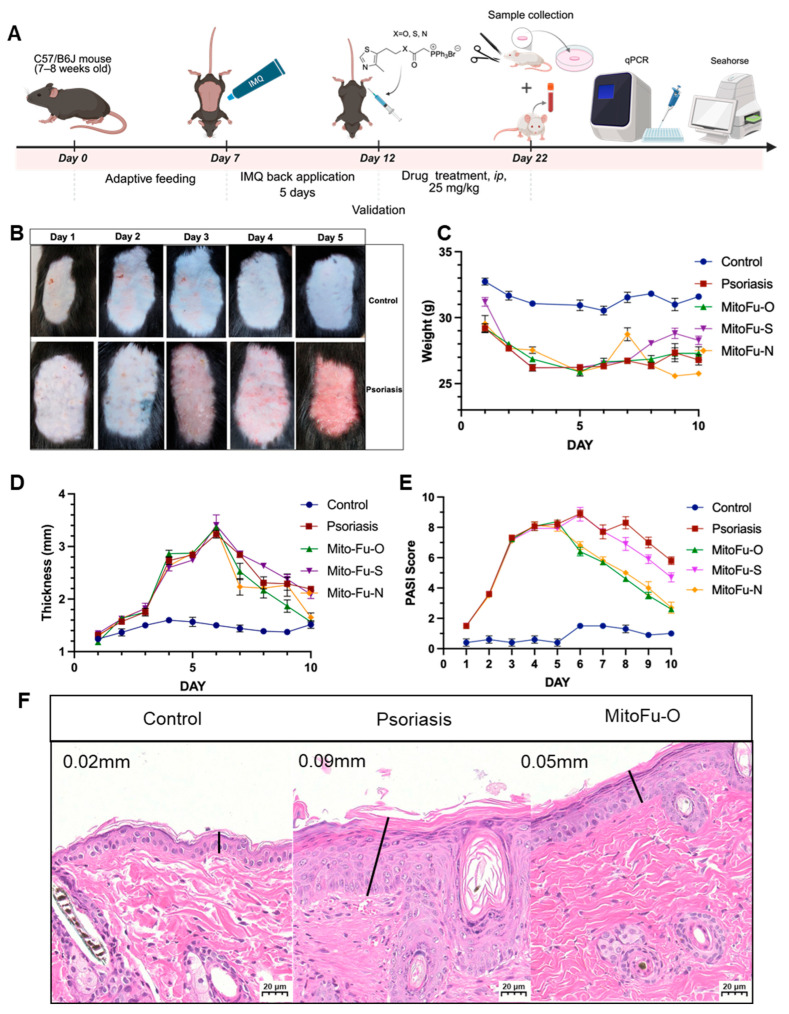
Effects of MitoFu derivatives in the imiquimod (IMQ)-induced psoriasis mouse model. (**A**) Schematic illustration of the experimental workflow of the IMQ-induced psoriasis mouse model. (**B**) Representative images of the mouse skin during the modelling. (**C**) Mouse body weight change. (**D**) Mouse skin sickness changes in different groups. (**E**) PASI scores in different groups. (**F**) Representative histological images of mouse skin sections stained with H&E. The bars indicate the magnification (20 μm). N = 6 mice per group.

To comprehensively evaluate the therapeutic potential of the MitoFu series, an IMQ-induced psoriasis-like mouse model was established, and mice were treated via intraperitoneal injection of each compound (Figure 3A). Following five consecutive days of IMQ topical application, the dorsal skin of the mice exhibited progressive erythema, scaling, and thickening (Figure 3B), confirming the successful induction of the model. By contrast, the MitoFu-O-treated group showed milder erythema and reduced desquamation, with a smoother skin surface by Day 5, suggesting that MitoFu-O effectively suppressed the development of psoriatic lesions (Figure 3B).

During the treatment phase, IMQ exposure showed mild but consistent weight loss (Figure 3C). Remarkably, MitoFu-O significantly prevented this decline, maintaining body weight close to baseline, which indicates favorable systemic tolerability and minimal toxicity. MitoFu-S exhibited partial efficacy without clear weight recovery, while MitoFu-N treatment resulted in transient systemic toxicity and subsequent treatment discontinuation. The toxicity was based on qualitative observations from preliminary experiments; for example, the mice suffered a sharp body temperature decrease and died before the modeling protocol was completed.

Quantitative analysis of skin thickness further highlighted the superior anti-psoriatic efficacy of MitoFu-O (Figure 3D). IMQ-induced mice displayed a more than two-fold increase in epidermal thickness, while MitoFu-O markedly reversed this hyperplasia, restoring skin thickness to near-control levels by Day 5. The other derivatives produced weaker or inconsistent improvement. Consistent with these observations, the Psoriasis Area and Severity Index (PASI) scoring (Figure 3E) showed that MitoFu-O induced a rapid and sustained reduction in erythema, scaling, and thickening, reaching almost normal levels by Day 10, whereas MitoFu-S and MitoFu-N achieved mild or temporary improvements.

Histological evaluation using hematoxylin and eosin (H&E) staining provided further confirmation (Figure 3F). IMQ-treated skin sections displayed marked epidermal acanthosis, parakeratosis, elongated rete ridges, and loss of the granular layer, all hallmarks of psoriatic pathology. Treatment with MitoFu-O notably normalized epidermal architecture, reducing keratinocyte proliferation, restoring the granular layer, and re-establishing regular keratinization patterns.

Collectively, these data (Figure 3A–F) demonstrate that MitoFu-O effectively mitigates IMQ-induced psoriatic inflammation and epidermal hyperplasia, outperforming its analogues with superior therapeutic efficacy and safety in vivo.

### 2.3. MitoFu-O, S, N Reduces Inflammatory Responses in IMQ-Induced Mouse Model

To further validate the effects of each drug on the inflammatory response in psoriasis, RT-qPCR was performed on the damaged skin tissues and whole blood samples of mice in each group after 5 days of treatment.

**Figure 4:**
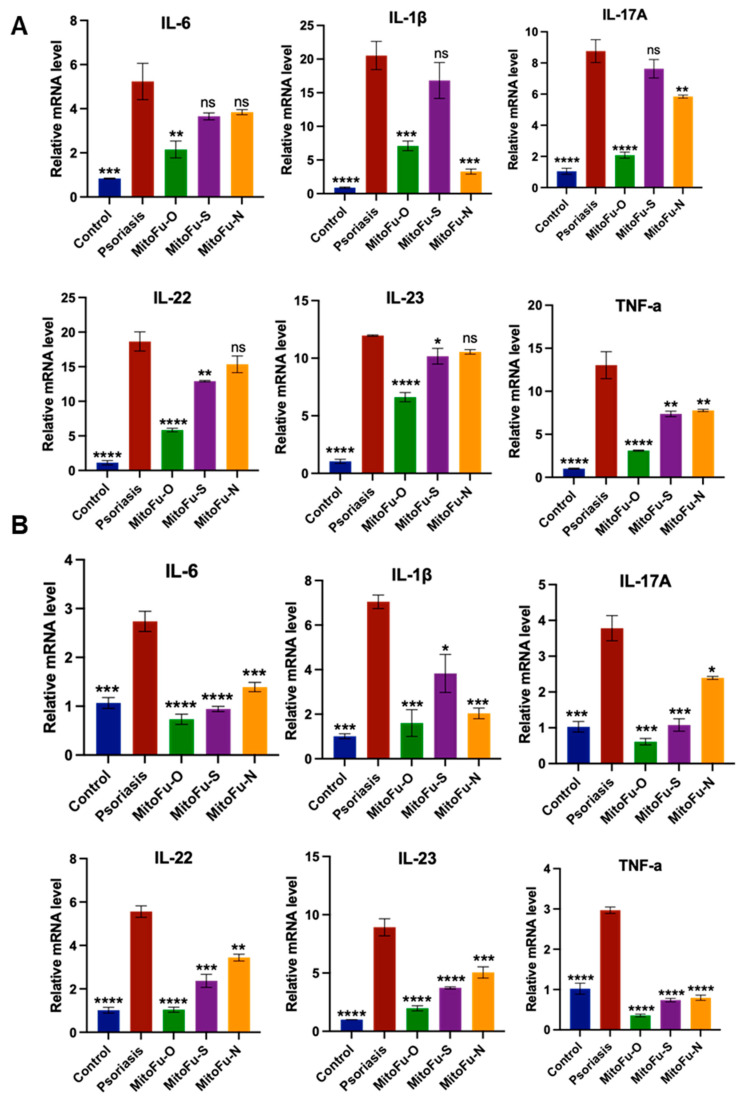
RT-qPCR analysis of mRNA levels of the proinflammatory cytokines in mouse tissues (**A**) and mouse whole blood (**B**) after 5 days of treatment. N = 6 mice per group. Data are mean ± SEM, * *p* < 0.05, ** *p* < 0.01, *** *p* < 0.001, and **** *p* < 0.0001, ns = not significant.

The expression of inflammatory factors in mouse tissues in the MitoFu-O group was significantly downregulated compared with the model group (*p* < 0.0001) (Figure 4A), indicating a strong anti-inflammatory effect. The MitoFu-S and MitoFu-N groups also exhibited a trend of downregulating inflammatory factor expression, although the downregulation was less pronounced, suggesting that their anti-inflammatory effects were relatively weak. Meanwhile, since MitoFu-N showed obvious toxic reactions during the administration, and further medication was discontinued, its therapeutic effect was not informative and was not considered further.

Additionally, the RT-qPCR results of whole blood samples (Figure 4B) showed a trend of consistency with those of the skin tissues. The expression of inflammatory factors was significantly reduced in mouse blood in the MitoFu-O group, suggesting that the drug not only exerts anti-inflammatory effects in local skin tissues but also has the potential to modulate systemic inflammatory responses.

In summary, the results of RT-qPCR analysis further corroborate the anti-inflammatory mechanism of MitoFu-O in the mouse model of psoriasis, which rapidly inhibits the expression of inflammatory factors and attenuates the immune response at an early stage, thereby achieving effective relief of pathological symptoms. MitoFu-O outperformed other derivatives in terms of inflammatory factors, demonstrating good therapeutic potential and promising translational prospects.

### 2.4. MitoFu-O Inhibits Aerobic Glycolysis in Psoriatic Cells and Mice

**Figure 5:**
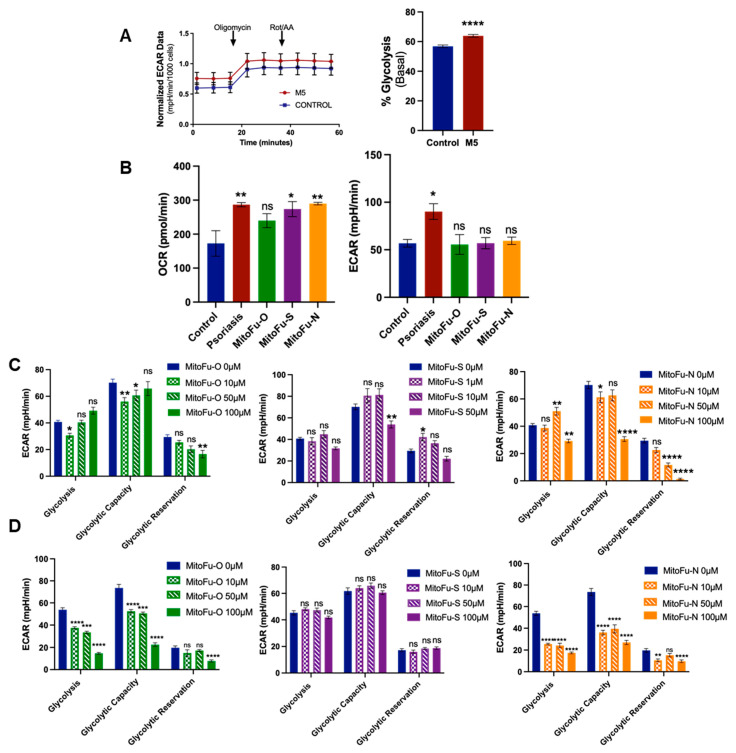
MitoFu-O reprograms glycolytic metabolism. (**A**) Real-time extracellular acidification rate (ECAR) analysis (N = 3). (**B**) Basal oxygen consumption rate (OCR) and basal ECAR of mouse skin tissues (N = 3 mice). (**C**,**D**) Dose-dependent effects of MitoFu-O, S, N on glycolysis, glycolytic capacity, and glycolytic reserve (N = 3) in Jurkat cell line (**C**) and CCRF-CEM cell line (**D**). * *p* < 0.05, ** *p* < 0.01, *** *p* < 0.001, and **** *p* < 0.0001, ns = not significant.

To investigate the impact of TPP-thiazole derivatives on cellular metabolism, an M5-treated HaCaT cell model was established. Seahorse XF ATP Rate Assay and Mito Stress Test were employed to assess mitochondrial function and glycolytic activity. As shown in Figure 5A, M5-treated cells exhibited a marked increase in extracellular acidification rate (ECAR), reflecting enhanced glycolytic activity.

To assess the effects of MitoFu-O on the metabolic reprogramming of psoriatic mouse skin, basal mitochondrial respiration and glycolytic flux were evaluated using the Seahorse XF Analyzer (Figure 5B). In the IMQ-induced psoriasis model, ECAR was markedly elevated compared with the control group, indicating a hypermetabolic state characterized by enhanced glycolysis. Treatment with MitoFu-O for five consecutive days significantly reduced basal ECAR levels compared to the model group, suggesting that glycolytic activity was suppressed. The Oxygen Consumption Rate (OCR) displayed a similar trend, but was less pronounced than ECAR. These findings indicate that MitoFu-O treatment counteracts the hypermetabolic state of psoriatic skin by primarily suppressing pathological glycolysis and, to a lesser extent, impacting mitochondrial respiration.

### 2.5. MitoFu-O Inhibits Aerobic Glycolysis in T Cell Lines

To evaluate the impact of the three compounds on T-cell glycolysis, Jurkat cells were treated for 24 h, and glycolytic function was assessed using the Seahorse XF Glycolysis Stress Test. The cell viability data (Figure S1) showed that IC_50_ of MitoFu-O, S, and N are all above 700 µM, showing no toxicity under 10–100 µM in the in vitro testing. As shown in Figure 5C, both MitoFu-O and MitoFu-N suppressed glycolysis in a concentration-dependent manner, whereas MitoFu-S showed no significant effects at any tested concentration. Specifically, at the highest tested concentration of 100 μM, MitoFu-O and MitoFu-N significantly reduced basal glycolysis to 27% and 32% of the control level, respectively. Additionally, glycolytic capacity was also significantly impaired; treatment with 100 μM MitoFu-O and MitoFu-N resulted in reductions of approximately 68% and 63%, respectively. Inhibition of the glycolytic reserve was pronounced only at the highest concentration of 100 μM.

A parallel assessment in CCRF-CEM cells revealed a more divergent response (Figure 5D). MitoFu-N exhibited the most potent inhibitory effects. At a concentration of 100 μM, it nearly ablated the glycolytic reserve and significantly suppressed both basal glycolysis and glycolytic capacity. In contrast, the inhibitory effect of MitoFu-O was weaker, showing approximately 25% inhibition of basal glycolysis and glycolytic capacity only at 10 μM, with no significant effects at 50 or 100 μM. Consistent with the results in Jurkat cells, MitoFu-S demonstrated no significant inhibitory effects in CCRF-CEM cells.

### 2.6. MitoFu-O Reprograms Glycolytic Metabolism Through Inhibition of Key Enzymes of Glycolysis

**Figure 6:**
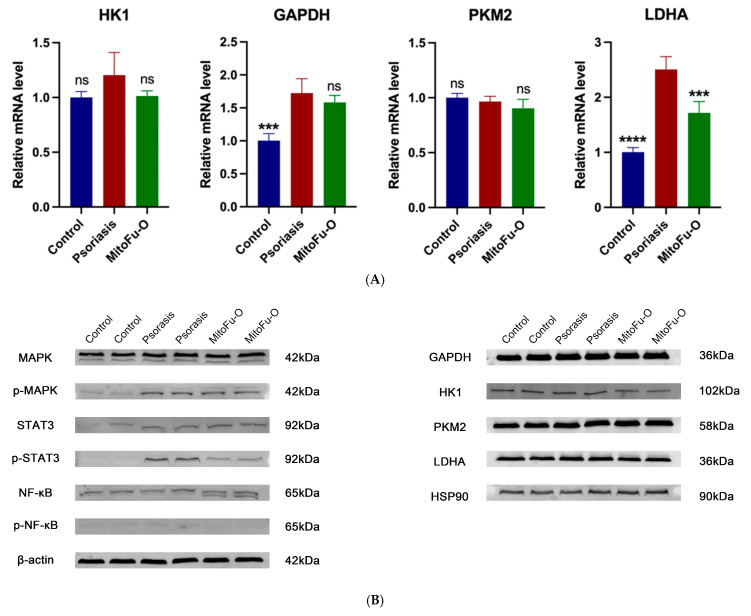
MitoFu-O inhibits aerobic glycolysis and suppresses MAPK/STAT3/ NF-κB signaling in psoriatic keratinocytes. (**A**) Relative mRNA levels of glycolysis-related genes (GAPDH, Glut1, HK1, LDHA, and PKM2) in control, psoriatic, and 50 μM MitoFu-O treated groups (*n* = 3). (**B**) Representative immunoblot images showing protein expression of glycolytic enzymes and signaling markers (GAPDH, HK1, PKM2, LDHA, MAPK, p-MAPK, STAT3, and p-STAT3). β-actin and HSP90 are housekeeping proteins. *** *p* < 0.001, and **** *p* < 0.0001, ns = not significant.

Given the critical role of metabolic reprogramming in psoriatic pathogenesis, the effect of MitoFu-O on key glycolytic enzymes was investigated. Western blot analysis revealed a significant increase in Lactate Dehydrogenase A (LDHA) protein levels in M5-stimulated HaCaT cells compared to controls, which was subsequently reversed by 50 μM MitoFu-O treatment (Figure 6B). Consistent with this finding at the protein level, RT-qPCR analysis demonstrated that the mRNA expression of LDHA was also markedly upregulated in the model group and downregulated following 50 μM MitoFu-O intervention (Figure 6A). A similar trend of M5-induced upregulation and MitoFu-O-mediated suppression was observed at the transcriptional level for other glycolytic genes, including Hexokinase 1 (HK1) and Glyceraldehyde-3-Phosphate Dehydrogenase (GAPDH) (Figure 5D). The coordinated upregulation of these genes suggests increased glycolytic flux in diseased cells. The fact that MitoFu-O normalized the expression of both LDHA protein and key glycolytic transcripts strongly suggests that its mechanism of action involves transcriptional regulation of metabolic pathways, thereby counteracting the aberrant glycolytic activity in psoriatic keratinocytes.

### 2.7. MitoFu-O Suppresses MAPK/NF-κB/STAT3 Signaling in Psoriatic Keratinocytes

We next assessed whether the therapeutic effect of MitoFu-O was associated with the inhibition of key pro-inflammatory signaling pathways. Western blot analysis was performed to examine the phosphorylation levels of MAPK, NF-κB, and STAT3. Compared with the control group, a marked upregulation in the phosphorylation of these signaling molecules was observed following M5 stimulation, confirming the hyperactivation of these pathways in our psoriatic model. Crucially, treatment with 50 μM MitoFu-O significantly suppressed this enhanced phosphorylation (Figure 6A). These data demonstrate that the anti-psoriatic effect of MitoFu-O is also mediated through the concurrent inhibition of the MAPK, NF-κB, and STAT3 signaling cascades.

## 3. Discussion

The central role of immune dysregulation, particularly the IL-23/Th17 axis, in psoriasis pathogenesis is well established, which has led to the successful development of biologic therapies [16,17]. However, the persistent clinical challenges of cost, variable response, and safety concerns underscore the need for alternative treatment strategies that target upstream, convergent disease mechanisms. Our study introduces a novel therapeutic paradigm for psoriasis by demonstrating that targeted modulation of mitochondrial metabolism, specifically by down-regulating aerobic glycolysis with a novel TPP-thiazole derivative, MitoFu-O, potentially alleviates psoriasiform inflammation in vitro and in vivo.

The rationale for targeting mitochondria stems from their emerging role as hubs integrating metabolic and inflammatory signals in psoriasis [12]. We posited that our mitochondria-targeting TPP-thiazole compounds, engineered for organelle-specific accumulation, could break the cycle of mitochondrial dysfunction, oxidative stress, and chronic inflammation. Among the derivatives tested, MitoFu-O emerged as the lead compound, demonstrating superior efficacy and a favorable safety profile. It significantly ameliorated disease severity in the IMQ-induced mouse model, as evidenced by reduced PASI scores, normalized epidermal architecture, and prevention of systemic weight loss—a key indicator of its tolerability compared to the transient toxicity observed with MitoFu-N.

**Figure 7:**
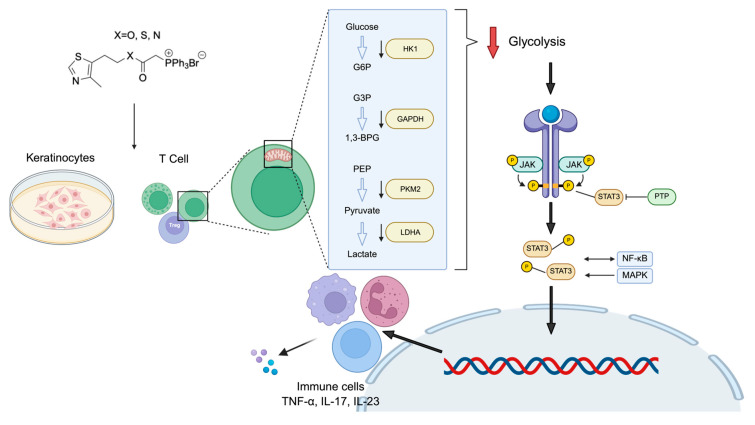
MitoFu-O inhibits aerobic glycolysis in keratinocytes and immune cells, exerting anti-inflammatory effects through NF-κB and STAT3 signaling cascades. The left panel depicts the canonical glycolytic cascade: Glucose is phosphorylated by hexokinase 1 (HK1) to glucose-6-phosphate (G6P), which proceeds through later steps to produce glyceraldehyde-3-phosphate (G3P). Glyceraldehyde-3-phosphate dehydrogenase (GAPDH) then catalyzes the formation of 1,3-bisphosphoglycerate (1,3-BPG), eventually leading to phosphoenolpyruvate (PEP). Pyruvate kinase M2 (PKM2) converts PEP to pyruvate, which is subsequently reduced to lactate by lactate dehydrogenase A (LDHA). The right panel represents potential upstream signaling pathways and regulatory nodes that may interact with or modulate glycolytic activity, including JAK-STAT3, PTP, NF-κB, and MAPK signaling cascades.

A pivotal finding of our work is the identification of aerobic glycolysis as a critical metabolic vulnerability in psoriatic pathology and the primary mechanism of action for MitoFu-O (Figure 7). The “Warburg-like” metabolic shift towards glycolysis, even in the presence of oxygen, is a recognized feature of hyperproliferating cells, including cancer and activated immune cells [27,28,29,30]. We extend this concept to psoriatic keratinocytes, showing that the inflammatory M5 cytokine cocktail induces a robust glycolytic phenotype, characterized by elevated ECAR. Crucially, MitoFu-O treatment reversed this hypermetabolic state, significantly suppressing glycolytic flux in both keratinocytes and T cells. This metabolic reprogramming was not a generic cytotoxic effect but a specific action, as MitoFu-O concurrently normalized the expression of key glycolytic enzymes, including HK1, GAPDH, and LDHA, at the transcriptional and protein levels. By dampening this pathological energy pathway, MitoFu-O likely deprives hyperactive keratinocytes and immune cells of the necessary biosynthetic precursors and energy for proliferation and inflammatory mediator production.

The anti-inflammatory effects of MitoFu-O are likely mediated through a dual mechanism: direct metabolic inhibition and subsequent suppression of key pro-inflammatory signaling cascades. We found that MitoFu-O significantly inhibited the phosphorylation of MAPK, NF-κB, and STAT3, three master regulators of inflammation, cell survival, and proliferation in psoriasis [31,32]. This finding is significant because it links metabolic reprogramming to inflammatory signaling. Enhanced glycolysis can promote inflammation through various mechanisms, including the production of lactate, which can stabilize HIF-1α and fuel NF-κB signaling [33]. By inhibiting glycolysis, MitoFu-O may disrupt this feed-forward loop, leading to the downstream deactivation of these critical pathways. This positions MitoFu-O as a multi-pathway inhibitor whose action originates at the metabolic level, offering a strategic advantage over agents that target single cytokines.

Glycolysis inhibition suppresses inflammation by disrupting the metabolic reprogramming of activated immune cells such as macrophages and T cells [28]. Under pro-inflammatory conditions, these cells rely on aerobic glycolysis to fuel cytokine production and proliferation. Blocking glycolysis via MitoFu-O cuts off energy supply, reduces pro-inflammatory cytokines (such as IL-1β, TNF-α), and may shift macrophages toward an anti-inflammatory state or increase the proportion of Treg cells that release anti-inflammatory cytokines. This effect is mediated through pathways like HIF-1α and mTOR, which link metabolism to immune function [27].

This work has several limitations. A positive reference compound was not included in the current study. Since the primary aim was to test whether our TPP-thiazole derivatives alleviate psoriasis, we focused on baseline comparison against a negative control to identify active hits rather than quantifying potency against a standard drug. Nonetheless, a positive control would strengthen future studies. While the IMQ model is a robust and widely accepted model of psoriatic inflammation, it does not capture the chronic, recurrent nature of human psoriasis, limiting its applicability to long-term disease progression. Other chronic models would be valuable. The structural differences among MitoFu-O, S, and N primarily lie in the heteroatom connected to the carbonyl group in the linker between TPP and the thiazole moiety, which results in different pharmacokinetic profiles of the compounds and further affects the pharmaceutical effects. Further research on pharmacokinetic studies can rationalize the different results obtained from MitoFu-O, S, and N. Additionally, while our data show a strong correlation between glycolysis inhibition and anti-inflammatory effects, further research using metabolic rescue experiments could provide more direct evidence of causality. Planned studies include (1) metabolite supplementation (e.g., pyruvate or lactate) in glycolysis-inhibited immune cells, which can test whether restoring glycolytic intermediates reverses inflammatory suppression, confirming on-target effects; (2) genetic approaches, such as overexpressing or knocking out key glycolytic enzymes like hexokinase 2 (HK2) or pyruvate kinase M2 (PKM2), which can validate the necessity of glycolysis for inflammation; (3) metabolic controls, including culturing cells in galactose to force oxidative phosphorylation or measuring glycolytic flux via extracellular acidification rate (ECAR), which should be used to link glycolysis directly to cytokine production; (4) modulating downstream pathways like HIF-1α or mTOR, which can determine if glycolysis inhibition acts through these signaling nodes.

## 4. Materials and Methods

### 4.1. HaCaT Cells and M5 Model

The HaCaT cell line was purchased from Fuheng Biology (Shanghai, China). M5 cytokine mixture (IL-22, IL-17A, oncostatin M, IL-1α, and TNF-α) was purchased from Thermo Fisher Scientific (Shanghai, China). These experiments were performed in the range of cell passages 5–14.

A measure of 25 g of each compound (MitoFu-O, S, N, white powder) was synthesized according to our previous publication [25]. All the compounds in this project came from the same batch. All compounds have a purity of over 98%.

HaCaT cells were cultured in Dulbecco’s modified Eagle’s medium (DMEM; Biosharp, Beijing, China) supplemented with 10% fetal bovine serum (FBS; Thermo Fisher Scientific) and 1% penicillin-streptomycin solution. HaCaT cells were cultured at 37 °C and 5% CO_2_ with humidity. Configured medium was stored at 4 °C and pre-warmed in a 37 °C water bath before subsequent use. Medium was changed every 1 to 2 days. Cells were sub-cultured after growing to about 70% confluence. The psoriasis cell model was established with 10 ng/mL of M5. The M5 powdered mixture was appropriately diluted with PBS and dissolved in DMEM. HaCaT cells were cultured in DMEM at 60–70% confluence and subsequently activated with M5 for 24 h.

### 4.2. IMQ-Induced Psoriatic Mouse Model

All animal procedures were reviewed and approved by the CAM-SU Animal Care and Use Committee of Soochow University (Approval No. LF-23-01, LF-24-01), and all experiments were conducted in compliance with the protocol #10124.

To evaluate the therapeutic effect of TPP-thiazole derivatives, 8-week-old male C57/B6J mice (GemPharmatech, Nanjing, China) were chosen and divided into five groups: control, model, MitoFu-O, MitoFu-N, and MitoFu-S (N = 6 mice per group). Mice in the model and treatment groups were given 62.5 mg of IMQ cream (5%, Mingxin, Chengdu, China) [34], The control group received the same amount of white Vaseline (Hai’s Hainuo, Qingdao, China) at the shaved 2 × 3 cm areas on the backs of the mice for 5 consecutive days, and the skin was observed for the appearance of erythema, desquamation, thickening, and peeling of the skin and was used for clinical psoriasis based on the Clinical Psoriasis Area and Severity Index (PASI) [35] scoring system to score skin inflammation on a daily basis.

After 5 consecutive days of successful modelling, the treatment was started on the sixth day, and the administered groups (MitoFu-O, MitoFu-S, MitoFu-N) were injected intraperitoneally at a dose of 25 mg/kg/0.3 mL per group (*n* = 6 mice per group), and the treatment was administered for 10 days. The purity of all compounds (MitoFu-O, MitoFu-S, MitoFu-N) was 99.9%+.

The PASI scoring for mouse skin was determined by evaluating erythema, scaling, and skin thickness, using a scale from 0 to 4, where 0 indicates none, 1 indicates mild, 2 moderate, 3 pronounced, and 4 severe manifestation [36]. Two independent dermatologists, blinded to the experimental conditions, evaluated and assigned the scores [37].

Mouse whole bloods were collected via retro-orbital blood collection under anesthesia at each time point for subsequent RT-qPCR and Western blotting. Mouse skin tissues (1.5 cm × 1.5 cm) were collected using surgical scissors for subsequent RT-qPCR, Western blotting, H&E staining, and Seahorse tests.

### 4.3. T Cells and Cell Viability Assay

The Jurkat cell was kindly provided by Dr Jinyuan Ho from Xi’an Jiaotong Liverpool University. The CCRF-CEM cell line was purchased from Fuheng Biology (Shanghai, China). Jurkat and CCRF-CEM cells were cultured in RPMI-1640 medium (Gibco, Shanghai, China) supplemented with 10% FBS (Thermo Fisher Scientific) and 1% penicillin-streptomycin solution. Jurkat and CCRF-CEM cells were cultured at 37 °C and 5% CO_2_ with humidity.

Jurkat cells were seeded in 96-well plates at a density of 2 × 10^4^ cells per well and cultured for 24 h at 37 °C in 5% CO_2_. Prior to treatment, cell density and uniform distribution were confirmed by microscopy to ensure consistent experimental conditions. The cells were then treated with varying concentrations (1 nM to 1 mM) of MitoFu-O, MitoFu-S, or MitoFu-N for 24 h, while the control cells were cultured in complete medium without drug treatment. Cell viability was assessed using the Cell Counting Kit-8. CCK-8 solution (cat. no. GK10001; Gipbio, Shanghai, China) was mixed with culture medium at a 1:10 ratio in each well. After 3 h of incubation, absorbance was measured at 450 nm using a microplate reader. All experiments were performed in triplicate, and cell viability was normalized to untreated control cells.

### 4.4. RT-qPCR

Skin tissues were weighed, added to TRIzol reagent (Invitrogen^TM^, Carlsbad, CA, USA) and grinding beads (Servicebio, Wuhan, China), and placed in a tissue pyrolysis instrument (Service, Wuhan, China) for complete lysis. Whole blood from mice was collected in EDTA anticoagulation tubes (KWS Conviviality, Shijiazhuang, China) and then preserved by adding TRIzol reagent. Cell pellets were collected with TRIzol reagent. Total RNA was extracted using the DNA/RNA extraction kit (Vazyme, Nanjing, China). The isolated RNA was then reverse-transcribed into cDNA using the ReverTra Ace qPCR RT Master Mix with gDNA Remover kit (TOYOBO, Shanghai, China). RT-qPCR was subsequently performed using specific primers (listed in Table 1). Each 20 µL RT-qPCR reaction mixture consisted of 2 µL cDNA, 10 µL SYBR Green Realtime PCR Master Mix (TOYOBO, Shanghai, China), 0.8 µL each of forward and reverse primers (1.6 µL total), and 6.4 µL ddH_2_O. The amplification protocol on a Real-Time PCR System (Thermo Fisher, Singapore) included the following thermal cycling conditions: initial denaturation at 95 °C for 15 s, annealing at 60 °C for 1 min, repeated for 40 cycles, followed by a final denaturation at 95 °C for 1 min. Data analysis was conducted using the 2^−∆∆Ct^ method [38].

### 4.5. Seahorse Tests

#### 4.5.1. XF Mito Stress Assay

Mitochondrial function of mouse skin tissues was assessed using the Seahorse XF24 (Agilent Technologies, Santa Clara, CA, USA) and the XF Cellular Mitochondrial Stress Test Kit (Seahorse Bioscience, Billerica, MA, USA) [39]. Mouse dorsal skin samples were collected and thoroughly rinsed with PBS. Circular punches were used to obtain uniform tissue sections, which were then incubated in DMEM medium. For each well of the XF24 Islet Capture Microplate (Agilent), 400 μL of DMEM medium was added, after which the skin tissue punches were placed into the wells and covered with islet capture screens. An additional 100 μL of DMEM medium was then added to each well. To ensure the reproducibility and statistical reliability of the experimental results, each group of skin samples was placed into 2 to 3 wells.

Before initiating the assay, 200 μL of sterile double-distilled water was added to each well of the utility plate to hydrate the sensor cartridge. The cartridge was then placed in a CO_2_-free incubator at 37 °C and left overnight to allow full sensor hydration. The next day, the water was removed and replaced with XF Calibration Solution. The cartridge was subsequently incubated for 1 h at 37 °C in a CO_2_-free environment to complete the calibration process before use.

During the Mito Stress Test, after preparing tissue samples according to the dosing protocol, a series of metabolic inhibitors was sequentially injected into the XF24 Islet Capture Microplate: oligomycin (55 μL, 50 μM; an ATP synthase inhibitor), FCCP (60 μL, 10 μM; a mitochondrial uncoupler), and a combination of rotenone and antimycin A (65 μL, 50 μM; inhibitors of complexes I and III).

Wave Pro software10.2.1.4 analyzed the data and exported it to GraphPad Prism 9 to obtain graphs and bar charts, and performed one-way ANOVA for statistical analysis of multiple sets of data.

Mitochondrial function of T cell lines was assessed using the Seahorse XF PRO (Agilent Technologies) and the XF Cellular Mitochondrial Stress Test Kit (Seahorse Bioscience) with similar procedures.

#### 4.5.2. XF Glycolytic Rate Assay

Mouse dorsal skin tissue samples were processed, and the sensor cartridge was hydrated as described above. The Seahorse XF Glycolysis Rate Assay Kit was used with the XF24 Islet Capture Microplate.

During the assay, oligomycin (56 μL, 25 μM) and 2-deoxyglucose (2-DG, 62 μL, 500 mM) were sequentially injected for the induction of glycolysis, the inhibition of mitochondrial ATP synthesis to induce maximal glycolysis, and the inhibition of the glycolytic process, respectively. Extracellular acidification rate (ECAR) was recorded in real time for assessing basal glycolytic capacity, glycolytic capacity, and glycolytic reserve.

Wave Pro software analyzed the data and exported it to GraphPad Prism to obtain graphs and bar charts, and performed one-way ANOVA for statistical analysis of multiple sets of data.

This experimental design aims to comprehensively assess the changes in the respiratory capacity of drugs on mitochondria by controlling variables and accurate statistical analysis, while ensuring the standardization of experimental conditions and the reliability of the results to provide a scientific basis for the metabolic improvement and therapeutic potential of the drugs.

Mitochondrial function of T cell lines was assessed using the Seahorse XF PRO (Agilent Technologies) and the XF Glycolysis Rate Assay Kit (Seahorse Bioscience) with similar procedures.

### 4.6. Western Blot

Protein was separated using 4–20% gradient precast gels (GenScript, Nanjing, China) at a constant voltage of 150 V for 1 h. The protein was then transferred to a PVDF membrane at 0.22 A for 65 min. Primary antibodies included GAPDH (CST, 2118), β-Actin (CST, 3700), p-MAPK (Erk1/2, Thr202/Tyr204) (CST, 4370), MAPK (Erk1/2) (CST, 4695), STAT3 (CST, 9139), p-STAT3 (Tyr705) (CST, 9145), HK1 (CST, 2024), HSP90 (CST, 4877), PKM2 (CST, 4053), and LDHA (CST, 3582). Images were quantified using ImageJ2 software.

### 4.7. H&E Staining

Following euthanasia, skin tissues were collected and preserved in 4% formaldehyde [40]. The fixed samples were then submitted for histological processing and analysis by Servicebio (Wuhan, China). Upon completion, epidermal thickness was assessed using SlideViewer 2.2.1 software.

### 4.8. Statistical Analysis

All data are expressed as mean ± standard error of the mean (S.E.M.). For comparisons between two groups, two-tailed Student’s *t*-tests were performed. When analyzing differences among multiple groups, one-way ANOVA followed by Bonferroni post hoc testing was used. Statistical analyses were conducted using Prism 8 (version 8.2.1, macOS). A *p*-value less than 0.05 was considered statistically significant. Significance levels were denoted as follows: * *p* < 0.05, ** *p* < 0.01, *** *p* < 0.001, and **** *p* < 0.0001.

## 5. Conclusions

In conclusion, we have demonstrated that targeted mitochondrial modulation with MitoFu-O represents a potent and novel therapeutic strategy for psoriasis. By primarily inhibiting the pathological aerobic glycolysis that fuels the inflammatory and hyperproliferative state, MitoFu-O concurrently dampens multiple pro-inflammatory signaling pathways. This approach moves beyond the paradigm of cytokine blockade to address a fundamental upstream driver of disease: metabolic reprogramming. Our findings not only establish MitoFu-O as a promising lead compound but also validate cellular metabolism, and specifically mitochondrial–glycolytic cross-talk, as a fertile ground for the development of next-generation, holistic therapies for psoriasis and potentially other immune-mediated inflammatory diseases.

## Figures and Tables

**Table 1:** Primers Sequences for RT-qPCR (m represents mouse, h represents human).

Primer	Forward	Reverse
GAPDH (m)	CAATGTGTCCGTCGTGGATCT	GTCCTCAGTGTAGCCCAAGAT
IL-6 (m)	CTGCAAGAGACTTCCATCCAG	AGTGGTATAGACAGGTCTGTTGG
IL-1β (m)	TTCAGGCAGGCAGTATCACTC	GAAGGTCCACGGGAAAGACAC
IL-17A (m)	TCAGCGTGTCCAAACACTGAG	CGCCAAGGGAGTTAAAGACTT
IL-22 (m)	ATGAGTTTTTCCCTTATGGGGAC	GCTGGAAGTTGGACACCTCAA
IL-23 (m)	CAGCAGCTCTCTCGGAATCTC	TGGATACGGGGCACATTATTTTT
SLC2A1 (h)	GGCTTCTCCAACTGGACCTC	CCGGAAGCGATCTCATCGAA
HK1 (h)	CACCAGTGATGTGTCAGCCA	GACAATGGTGCAAACGTGCT
PKM (h)	GGAAGCCTGTCATCTGTGCT	CCTCACGAGCTATCAGGTGC
LDHA (h)	TTGTCTCTGGCAAAGTGGAT	CTCCATGTTCCCCAAGGACC
GAPDH (h)	AATGGGCAGCCGTTAGGAAA	GCGCCCAATACGACCAAATC
IL-6 (h)	AGCGCCTTCGGTCCAGTTGC	GTGGCTGTCTGTGTGGGGCG
TNF-α (h)	ATGAGCACTGAAAGCATGATCC	GAGGGCTGATTAGAGAGAGGTC
β-actin (h)	GTGCTATCCCTGTACGCCTC	CGGACTCGTCATACTCCTGC
IL-8 (h)	CTCCAAACCTTTCCACCCCA	TTCTCAGCCCTCTTCAAAAACT

## Data Availability

Data will be made available on request.

## Supplementary Materials

The following supporting information can be downloaded at: https://www.mdpi.com/article/10.3390/molecules31060982/s1, Figure S1: Cytotoxicity assay of MitoFu-O, S, N on Jurkat cell lines via CCK8 assays.

## Abbreviations

The following abbreviations are used in this manuscript:

ECAR	Extracellular Acidification Rate
GAPDH	Glyceraldehyde-3-Phosphate Dehydrogenase
H&E	Hematoxylin and Eosin
HK1	Hexokinase 1
IMQ	Imiquimod
LDHA	Lactate Dehydrogenase A
OCR	Oxygen Consumption Rate
PASI	Psoriasis Area and Severity Index
PKM2	Pyruvate kinase M2
TPP	Triphenylphosphonium
