# Supplementary material for: TPP-Thiazole Derivatives Ameliorate Psoriasiform Inflammation by Glycolysis Inhibition
Source: Molecules. 2026 Mar 15;31(6):982. doi: 10.3390/molecules31060982 (PMC13029098; doi:10.3390/molecules31060982)
Supplement: Supplementary file 1 [file molecules-31-00982-s001.zip › molecules-4154903-supplementary.pdf]

Supplementary Information

TPP-Thiazole Derivatives Ameliorate Psoriasiform Inflammation by Glycolysis Inhibition

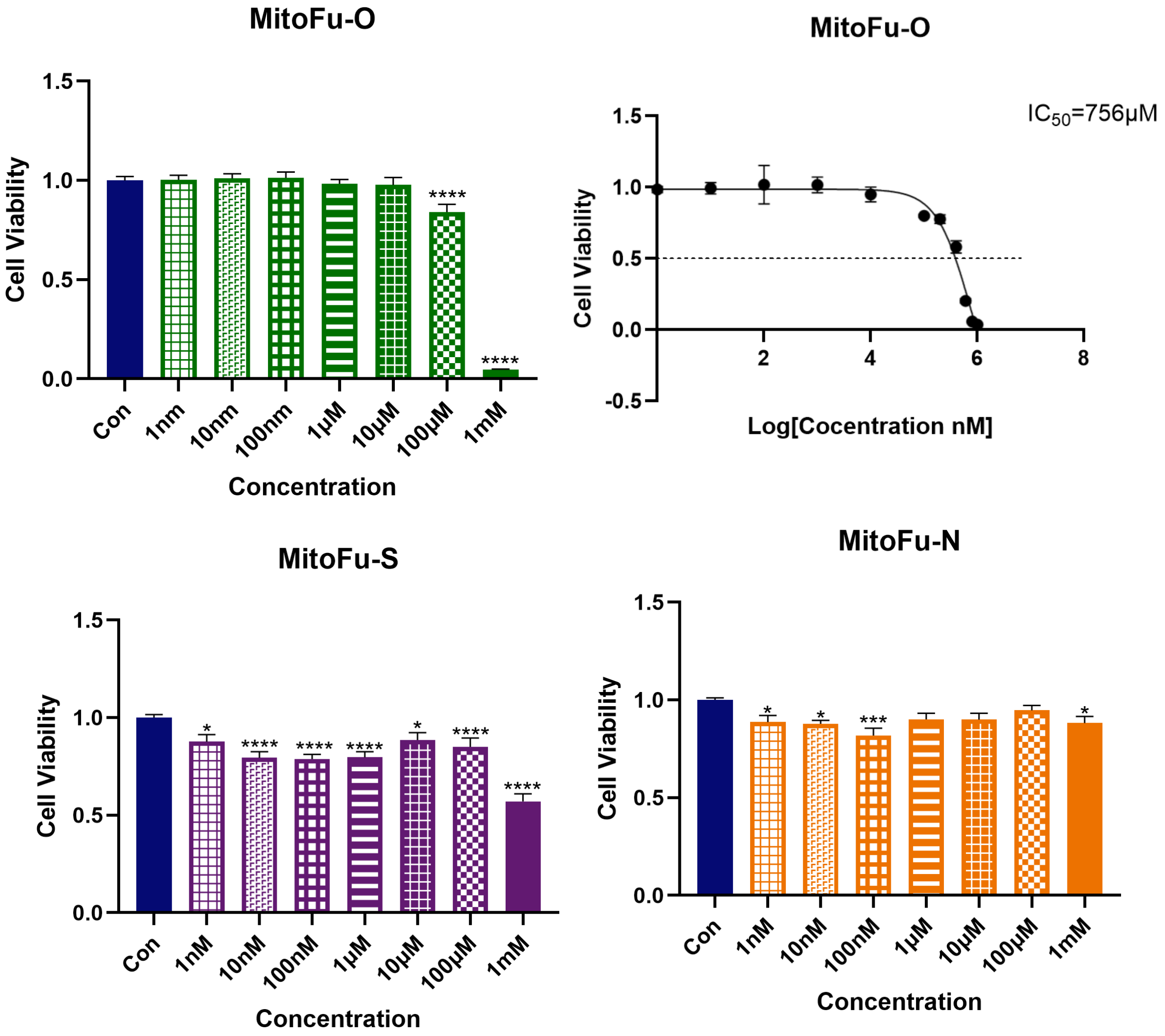

Figure S1. Cytotoxicity assay of MitoFu-O, S, and N on Jurkat cell lines via CCK8 assays. Jurkat cells were treated with three compounds at the indicated concentrations (1 nM–1 mM) for 24 h, while control cells were cultured in complete medium without drug treatment. The IC_{50} of MitoFu-O, S, and N are all above 700 µM, showing no toxicity under 50 ‑ 100 uM in in vitro testing. Data are presented as mean ± SEM from three independent biological experiments. Statistical analysis was performed using one-way analysis of variance (one-way ANOVA). *P < 0.05, **P < 0.01, ***P < 0.001, ****P < 0.0001 versus control.
